# The PIN-FORMED Auxin Efflux Carriers in Plants

**DOI:** 10.3390/ijms19092759

**Published:** 2018-09-14

**Authors:** Jing-Jing Zhou, Jie Luo

**Affiliations:** College of Horticulture and Forestry Science, Hubei Engineering Technology Research Center for Forestry Information, Huazhong Agricultural University, Wuhan 430070, China; hupodingxiangyu@mail.hzau.edu.cn

**Keywords:** auxin, function, *PIN* gene family, regulation, transporter

## Abstract

Auxin plays crucial roles in multiple developmental processes, such as embryogenesis, organogenesis, cell determination and division, as well as tropic responses. These processes are finely coordinated by the auxin, which requires the polar distribution of auxin within tissues and cells. The intercellular directionality of auxin flow is closely related to the asymmetric subcellular location of PIN-FORMED (PIN) auxin efflux transporters. All PIN proteins have a conserved structure with a central hydrophilic loop domain, which harbors several phosphosites targeted by a set of protein kinases. The activities of PIN proteins are finely regulated by diverse endogenous and exogenous stimuli at multiple layers—including transcriptional and epigenetic levels, post-transcriptional modifications, subcellular trafficking, as well as PINs’ recycling and turnover—to facilitate the developmental processes in an auxin gradient-dependent manner. Here, the recent advances in the structure, evolution, regulation and functions of PIN proteins in plants will be discussed. The information provided by this review will shed new light on the asymmetric auxin-distribution-dependent development processes mediated by PIN transporters in plants.

## 1. Introduction

Auxin is a universal hormone in plants, which participates in many aspects of plant developmental and growth processes [1]. The actions of auxin were firstly described by Charles and Francis Darwin, father and son [2]. Afterwards, auxin was isolated and discovered by Went in 1928 [3,4]. Since then, much attention has been paid to understanding the pathways involved in its synthesis, translocation and signaling [1,5,6]. This hormone is unique to plants, which requires polar transport. Auxin is usually synthesized in the shoot apex, as well as the developing leaf primordia, and transported to the targeted tissues by bulk flow via vascular tissues or direct polar transport [7,8]. Thus, to facilitate polar auxin transport (PAT), several auxin transporters were identified, such as AUXIN1/LIKE-AUX1 (AUX/LAX), PIN-FORMED (PIN), ATP-binding cassette (ABC) transporters, nitrate transporter 1.1 (NRT1.1), PIN-Like transporters (PILS) and WALLS ARE THIN 1 (WAT1) [7,9,10,11,12,13,14,15,16,17]. These auxin carriers can be generally divided into two groups based on the subcellular locations and functions. One group (comprised of AUX/LAX, NRT1.1, ABC transporters and most PIN) is located on the plasma membrane (PM) as import or export carriers to facilitate auxin distribution within cells. The other group (comprised of several PIN with short hydrophilic loops, PILS and WAT1) is distributed on the intracellular compartments to regulate the intracellular auxin homeostasis [6,12,14]. With the help of these auxin carriers, the auxin can be transported in a polar manner within cells, where specific maxima and minima levels of auxin can be sensed by the downstream regulation modules, such as the TRANSPORT INHIBITOR RESPONSE 1/AUXIN SIGNALING F-BOX (TIR1/AFB)-Auxin/INDOLE-3-ACETIC ACID (Aux/IAA)-AUXIN RESPONSE FACTOR (ARF) signal pathway, to transform endogenous and exogenous stimuli into gene reprogramming events [5,18,19].

Among the mentioned auxin carriers above, the polar localizations of PIN proteins finely correspond to the directionality of auxin movement, which highlights that PIN proteins are mainly responsible for the asymmetric distributions of auxin in plants [7,8,20]. The polar localizations of PIN are fine-tuned by a set of regulators that phosphorylate their different phosphosites on the central long hydrophilic loop (HL) [6]. The phosphorylated PIN proteins dynamically localize on the PM, which is involved in PIN’s subcellular trafficking, recycling, as well as turnover in a ubiquitination-dependent manner [21]. In recent years, several excellent review papers nicely summarized the evolution, polar localization and regulation of PIN proteins in plants [6,21,22,23]. In this review, we will briefly summarize the recent advances on the identification, molecular structure, evolution, co-expression and protein interactions, regulation and functions of PIN proteins in plants. The information provided by this review will shed new light on the molecular mechanisms of the auxin maxima mediated by the activities of PIN efflux carriers, as well as the roles of PIN proteins in regulating plant development and growth.

## 2. The Identification and Molecular Structure of PIN Proteins in Plants

The PIN gene family encodes a subgroup of auxin efflux carriers, which costs energy from the electrochemical gradient across the PM [24]. *PIN1* in *Arabidopsis* was the first identified *PIN* gene, afterwards, seven additional *PIN* genes were found in the genome of *Arabidopsis* [17,21,25]. To date, *PIN* genes have been identified in 31 plant species by genome-wide approaches, including *Brassica rapa*, *Glycine max*, *Medicago truncatula*, *Oryza sativa*, *Populus trichocarpa* and *Zea mays* (Table 1). The number of *PIN* genes ranged from 4–23, and the lowest and highest numbers of *PIN* genes were found in *Marchantia polymorpha* and *Glycine max*, respectively (Table 1) [26,27]. Although the number of *PIN* genes does not significantly correlate with the number of predicted loci (Table 1), genome duplication plays crucial roles in expanding the *PIN* gene family [26,27,28,29,30]. For instance, in *Glycine max*, the expanding number of *PIN* genes was closely linked with the two whole-genome duplication events, which was similar to the origins of other genes in this species, such as *Aux*/*IAA* genes [18,26]. Although the genome information of several gymnosperm plants has been already released in Phytozome (available online: https://phytozome.jgi.doe.gov/pz/portal.html) and other databases, the information about *PIN* genes in these gymnosperm plants is still very limited at a genome-wide scale (Table 1). In addition to the genome-wide analysis, many *PIN* members were partly identified in other plant species, such as *Malus domestica*, peach, *Cardamine hirsute* and algae [31,32,33,34,35,36]. The identification of *PIN* genes in diverse plant species accelerates the understanding of molecular structure and evolution history and also provides the fundamental information to be used for a comparative functional study of these *PIN* genes in plants.

**Figure 1:**
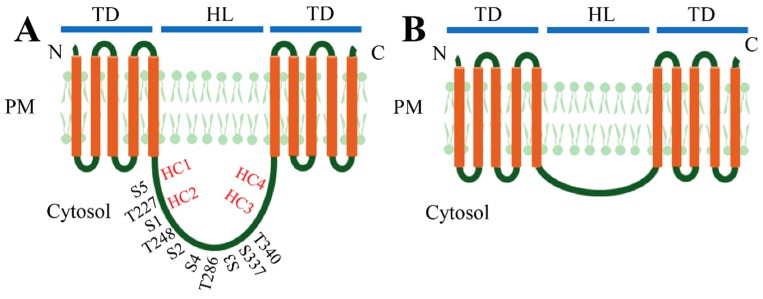
Molecular structures of (**A**) long PIN proteins (PIN1–4, 6 and 7) and (**B**) short PIN proteins (PIN 5 and 8). The PIN proteins harbor a typical central long hydrophilic loop (HL) between amino- and carboxy-terminal ends with five transmembrane domains (TD) spanning on the plasma membrane (PM). The phosphosites on the HL of long PINs are shown [6]. The conserved HC1–HC4 regions in the central loop domain are also indicated according to Bennett et al. [36].

The PIN carriers in plants participate in auxin transport at both intercellular and intracellular levels. The differences in function are dependent on the subcellular localizations of different PIN proteins with distinct molecular structures [58]. All the PIN proteins harbor a typically conserved HL with approximately 35 identifiable motifs located between amino- and carboxy-terminal transmembrane domains (TD), likely forming an auxin-translocation pore [17,22,59]. Based on the size of the central HL, the PIN proteins can be generally divided into two major subgroups, the “long” and “short” PIN proteins (Figure 1) [17]. In *Arabidopsis*, PIN1–PIN4, PIN6 and PIN7 are long PIN proteins. PIN5 and PIN8 are considered as short PIN proteins [17,29,59]. The long PIN proteins mainly localize at the PM, which facilitate the auxin fluxes within cells as auxin export carriers [17]. Some long PIN proteins with reduced “long HL”, such as PIN6 in *Arabidopsis*, are dual located at both the PM and endoplasmic reticulum (ER) depending on the phosphorylation states [60,61,62]. These PIN proteins have a distinct intracellular HL between amino- and carboxy-terminal ends with five TD spanning in the PM on each side of HL and the C-terminal end positions on the apoplast [17,59,63]. The short PIN proteins mainly localize at the ER with an inconspicuous central HL and contribute to the intercellular auxin homeostasis [59]. The PIN proteins contain many more conserved sites on the TD regions than on the HL, and the long PIN proteins in all plants can be further classified into seven subgroups according to the variants on the HL [17]. The HL also contains three conserved domains (C1–C3) and two variable domains (V1 and V2) [24]. Additionally, three conserved TPRXS sites containing T227/S1, T248/S2 and T286/S3, respectively (Figure 1), were found in the HL of long PIN proteins as the targets of the PINOID (PID) family kinases, D6 protein kinase (D6PK) and mitogen-activated protein kinase (MAPK) for phosphorylation of the serine or threonine residues. Two other serine residues (i.e., S4 and S5) also exist in this region (Figure 1A) [59,64,65,66]. These phosphorylation sites are essential for PIN’s polar localization and absent in the same regions of short ones [59]. In addition to phosphorylation sites, two evolutionarily-conserved cysteine residues (i.e., C39 and C560) on the TD were identified as *cis*-acting regulators to regulate PIN2 polar localization [67].

In addition to the terms “long” and “short” PIN proteins, another pair of more precise terms, “canonical” and “noncanonical” PIN proteins is proposed based on the existing highly-conserved HC1–HC4 regions in the central loop domain [36]. The PIN proteins with a long central loop usually harbor all four conserved motifs with high similarity; therefore, these PIN proteins are classified as canonical PIN proteins [36]. Accordingly, the remaining ones are noncanonical PIN proteins [36].

## 3. Genetic Evolution of PIN Proteins in Plants

The *PIN* genes have been suggested to evolve from a single ancestral gene, since *PIN* genes from higher plants shared relatively high sequence similarities with each other compared to the homologues of the closest bacteria [30]. The PIN proteins are also considered to be unique in land plants originating in streptophyte algae, which is the ancestor of land plants in plant evolutionary history [29,68]. The ER-based PIN proteins are proposed to be ancestral PINs within land plants, and the PM ones are acquired to coordinate the directional PAT in multicellular plants co-appearing with long PIN proteins during the later evolution of land plants [29,60,69]. However, this point of view has been challenged by a wider and deeper evolution analysis with 473 PIN family members, which suggests PIN proteins gained sub- and neo-functionalization by modifying the protein structure during plant evolution [22,36]. The canonical PIN proteins with a modular central loop domain are suggested to be ancestral ones with a more conserved structure, and the noncanonical ones with a divergent protein structure evolved from within a canonical lineage with neofunctionalization [22,36]. The motifs in the central loop domain can be targets of diverse regulators. Thus, selective modifications of some motifs in the intracellular loop contribute to the subfunctionalization of canonical PIN proteins [36,70]. Further evidence from PIN proteins’ evolutionary history indicate that PIN-mediated auxin transport only contains a few innovations in the central loop domain and has not contributed to the evolution of plant development [22,36]. Thus, it is the previously-evolved new developmental processes that have selected the structurally-divergent PIN proteins arising from the canonical precursors, rather than the opposite scenario [22,36].

## 4. Co-Expression Network and Protein Interactions of PIN Proteins in *Arabidopsis*

**Figure 2:**
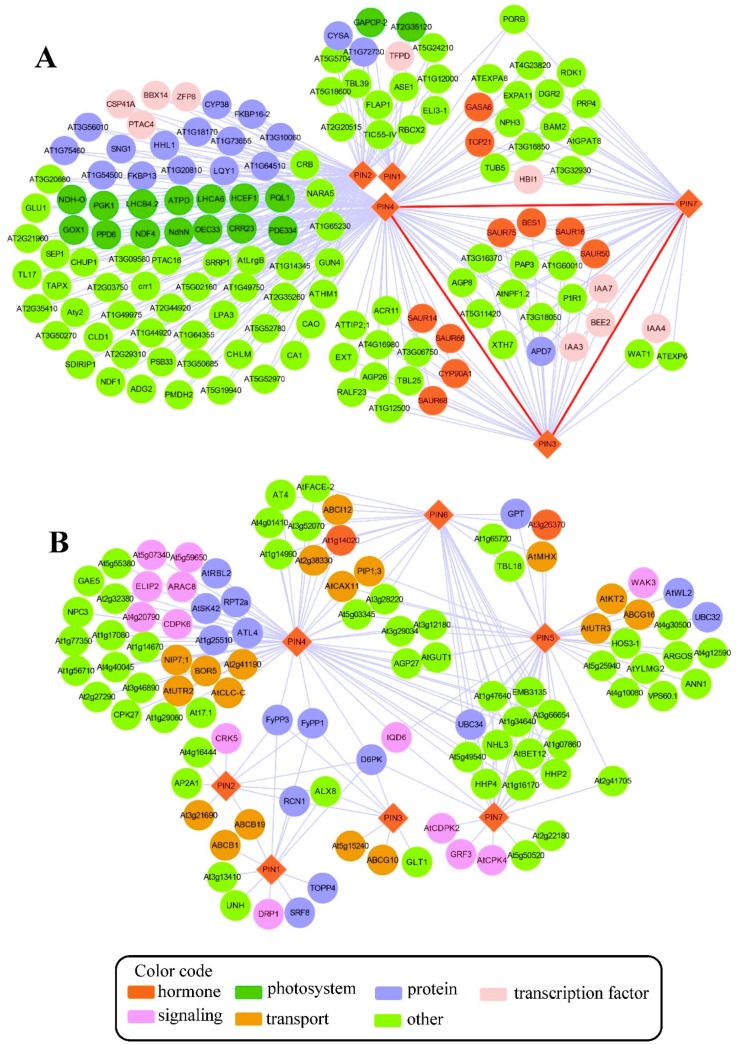
(**A**) The co-expression network of *PIN* genes and (**B**) the PIN protein interaction network in *Arabidopsis*. The network was constructed using an online database, CressExpress (available online: http://cressexpress.org/home.xhtml). This was done using pathway-level co-expression (PLC) analysis as suggested by Wei et al. [71] and visualized in Cytoscape (3.5.0) [86]. In the co-expression network, the lines between nodes represent significant correlations between two genes. The red lines represent significant correlations between *PIN* genes. The protein-protein interaction network was constructed using Cytoscape with data from the BioGRID database [87] (available online: https://thebiogrid.org/). In the PIN protein interaction network, the lines between nodes represent physical interactions verified by experimental approaches (for detail, see Table S1). The *PIN* genes/PIN proteins are represented by diamonds, and other co-expressed genes/interactive proteins are represented by circles. The different colors indicate different functional categories assigned by Mapman software [88], as shown by color codes.

Genes involved in the same pathways are highly co-expressed compared to genes from different pathways [71]. Thus, the co-expression analysis of a sub-set of genes and finding their co-expressed neighbors could be a powerful tool to dissecting candidate genes that potentially participated in a specific pathway [71,72,73]. Based on this notion, a pathway-level co-expression (PLC)-based *PIN* co-expression network was studied in *Arabidopsis* using an online database CressExpress (Version 4.0, available online: http://cressexpress.org/home.xhtml) with data from 8941 arrays [71]. In the PLC network, five of the eight *PIN* genes (e.g., *PIN1*, *PIN2*, *PIN3*, *PIN4* and *PIN7*) were co-expressed with another 141 genes via 303 correlations (Figure 2A). Interestingly, the *PIN* genes in the co-expressed network all belonged to the canonical group with a long central loop. This was related to the fact that the canonical proteins have a distinct cellular location and functions in auxin transport compared to the noncanonical ones. The direct significant correlations of *PIN* genes were only found among the *PIN3*, *PIN4* and *PIN7* genes. This confirmed that these three *PIN* genes share similar expression profiles and have a close evolutionary relationship [30]. Additionally, nine early auxin-responsive genes (six *small auxin-up RNA* (*SAUR*) and three *IAA*) identified in this network all belonged to the *PIN3*-*PIN4*-*PIN7* module, suggesting that the *PIN3*-*PIN4*-*PIN7* co-expression module plays crucial roles in auxin transport and signaling processes and that the two processes were finely co-regulated at the transcriptional level. Additionally, two genes encoding the basic helix-loop-helix (bHLH) protein, *HOMOLOG OF BEE2 INTERACTING WITH IBH 1* (*HBI1*) and *BR ENHANCED EXPRESSION 2* (*BEE2*), were found in the core *PIN3*-*PIN4*-*PIN7* module. These are known as regulators of cell elongation via targeting their downstream auxin metabolism relative genes [74,75]. Given the fact that these two genes were highly correlative with *PIN3*, *PIN4* and *PIN7*, it is of interest to check the specific roles of these two genes in PIN-mediated auxin efflux. Overall, the information obtained from the co-expression network conforms to the current knowledge on the regulation of *PIN* genes and also provides the possible routes to dissect the new candidates involved in PIN-mediated auxin efflux.

In addition to the co-expression network, by taking advantage of large-scale interactome analyses with diverse experimental approaches in *Arabidopsis*, it has become possible to identify a large set of physical interactors of PIN proteins (Figure 2B, Table S1). The comprehensive physical interaction map consisted of seven PIN proteins and 104 other proteins with 183 interactions (Figure 2B, Table S1). About 39% of the interactors shared interactions with at least two PIN proteins. Among the shared interactors, 12 interactors were commonly shared by four PIN proteins: PIN4, PIN5, PIN6 and PIN7. It is strange that no interactors were found for PIN8 in this interactome map, even after using some predicted methods (Figure 2B, Table S1). Many known interactive proteins relative to the functions of PIN proteins can be generally classified into three major categories in the PIN protein interaction network. One was related to regulating polar PIN distribution by phosphorylating the central loop of PIN proteins, including D6PK, Ca^2+^/calmodulin-dependent protein kinase-related kinase 5 (CRK5), type-1 protein phosphatase 4 (TOPP4), phytochrome-associated serine/threonine-protein phosphatase 1 (FyPP1), FyPP3, ROOTS CURL IN NPA1 (RCN1) and SAPS-domain-LIKE like 1 (SAL1) [76,77,78,79]. The other category of interactors participated in proper dynamic polar distribution of PIN proteins. This category included dynamin-related protein 1A (DRP1A), AP2A1 and UNHINGED (UNH) [80,81,82]. The last category contained four ABC transporters: ABCB1, ABCB19, ABCG10 and ABCG16. Evidence confirmed that ABCB and PIN proteins function interactively with independent auxin transport mechanisms, coordinating actions of the auxin export across PM depending on the plant tissues [13,83]. The remaining interactors in this network were basically identified by large-scale membrane protein interactome approaches [84,85]. The specific roles of these interactors in PIN proteins’ regulation need to be further validated. It is of interest that 14 transporters besides ABC transporters shared direct interaction with PIN proteins. These transporters were involved in transporting a broad variety of substrates, such as amino acids, calcium, water, metal, sugar, potassium, and so on (Figure 2B, Table S1). This implies the possible role of auxin polar distribution in transporting these substrates. Due to the limitation of this database, some well-known regulators, such as PID, were not included in this PIN interactive network. However, the identified interactors and their interactions in the interactome map could be a nice index to dissect the possible new mechanisms regulating the PIN proteins at the protein level.

Overall, these resources provided a range of candidates potentially involved in regulating PIN proteins at both transcriptional and protein levels. These candidates participate in multiple processes beyond plant development and growth. Thus, new roles of PIN proteins in these aspects may be uncovered by digging this valuable mine of information.

## 5. Regulation of PIN Proteins at Multiple Levels

Auxin polar transport facilitated by PIN proteins plays a pivotal role in determining the auxin maxima and minima within cells via stitching auxin metabolism together with signal transduction processes. The activities of PIN proteins are turned in multiple dimensions to convert the internal and external stimuli into down-stream gene reprogramming events in a rapid and precise manner. Much attention has been paid to dissecting the regulation of PIN proteins. These processes are involved in regulating PIN gene expression levels, post-transcriptional modifications of PIN proteins to facilitate their cellular trafficking, PIN proteins recycling and turnover [6,21,23,65,89,90,91,92,93].

### 5.1. Regulating PIN Genes Expression at the Transcriptional Level

The expression of *PIN* genes is finely regulated by diverse hormonal and environmental stimuli [17,21]. Most *PIN* genes are induced by auxin itself, except *PIN5*, the transcription of which is downregulated by auxin [17,60]. Additionally, a set of transcription factors act as up-stream factors targeting the promoter regions of *PIN* genes to regulate their expression levels in response to changes in endogenous and exogenous signals [90,91]. *XAANTAL2* (*XAL2*/*AGL14*), belonging to the MADS-box gene family, was the first identified transcriptional factor [94]. It directly binds to the promoters of *PIN1* and *PIN4* to upregulate their expression and is involved in auxin-mediated root developmental processes in a PIN4-dependent manner [94]. Furthermore, *PIN1* has been shown to be one target of INDETERMINATE-DOMAIN 16 (IDD16) [95]. IDD14–IDD16 cooperatively regulate the lateral organ patterning and gravitropism by enhancing the transcriptional levels of several genes involved in auxin biosynthesis and transport, such as *YUCCA5* (*YUC5*) and *PIN1* [95]. A plant-specific DNA binding protein with an unknown function, PIN2 PROMOTER BINDING PROTEIN 1 (PPP1), was identified as a *PIN2* regulator by the yeast one-hybrid methodology. In silico analysis revealed that parts of PPP1 DNA-binding sites commonly exist in the promoter regions of most *PIN* genes [96]. Recently, an Apetala2 (AP2) transcription factor, WRINKLED1 (WRI1), which is involved in fatty acid biosynthesis, has been show to directly target the promoters of *PIN4* and *PIN5* and cause reduced expression levels of some *PIN* genes (e.g., *PIN1*, *PIN3* and *PIN5*-*6*) in the *wri1-1* mutant background compared to wildtype specimens [97]. This study shed new light on the possible link between *PIN* gene expression and fatty acid synthesis.

The *Arabidopsis* R2R3-MYB transcription factor FOUR LIPS (FLP, MYB124), in cooperation with MYB88, targets the promoters of *PIN3* and *PIN7* genes to complementarily establish gravitropic set-point angles of lateral roots [98]. Further study showed that both *FLP* and *PIN3* are targets of auxin response factor 7 (ARF7) and that lateral root development is mediated by PIN3, in cooperation with both ARF7 and FLP [99]. Another ARF protein, MONOPTEROS (MP/ARF5), has also been shown to activate the expression of three *PIN* genes (namely *PIN1*, *PIN3* and *PIN7*) by targeting the auxin response elements (AuxRE) in the promoter regions of these genes to accomplish the patterning processes in both shoots and roots in an auxin-dependent way [91]. Interestingly, *PIN1*/*3*/*7* were also shown to be the direct targets of another ARF transcriptional factor ARF3/ETTIN [100]. These results collectively shed new light on integrating the auxin signaling and transport to the transcriptional regulation of PIN proteins to finely tune the developmental outcomes in plants [91]. In addition to being regulated by ARF, a group of cytokinin response factors (CRFs) (i.e., CRF2 and 6) acts as downstream cytokinin signaling, to activate the expression of both *PIN1* and *PIN7* via the specific PIN cytokinin response element (PCRE) located on the promoter regions of these two *PIN* genes [101]. Interestingly, *CRF2* itself is the target of MP/ARF5, which also binds to the promoters of CRF2′s targets, *PIN1* and *PIN7* [91,102]. Additionally, another bHLH transcription factor, SPATULA (SPT), which enables cytokinin signaling, activates the expression of *PIN3* to regulate young gynoecium growth [103]. Taken together, these results suggest the role of integrating cytokinin and auxin signaling on regulating auxin polar transport.

In addition, the SWI/SNF chromatin remodeling ATPase BRAHMA (BRM) acts as the upstream of the PLETHORA (PLT) pathway by directly targeting the chromatin of several *PIN* loci (i.e., *PIN1*–*4* and *7*), to facilitate the formation of the root stem cell niche [104]. The recruitment of BRM by MP/ARF5 to the MP/ARF5 target loci is essential to activating the chromatin state for auxin-response gene expression [5,105], together with the fact that several *PIN* genes (i.e., *PIN1*, *PIN3* and *PIN7*) are targeted by MP/ARF5 [91]. Thus, this could be another possible alternatively route for regulating *PIN* gene expression by BRM. These data indicate that chromatin remodeling of *PIN* genes plays a crucial role in regulating *PIN* expression levels.

### 5.2. Post-Transcriptional Modifications of PIN Proteins

In recent years, much progress has been made in understanding the activation and regulation of PIN proteins by phosphorylation, as well as the possible mechanisms of phosphorylation-mediated PIN subcellular trafficking [6,21,89,92,106,107,108,109]. Phosphorylation is essential for PIN polar distribution in the PM, which is coordinated by a set of protein kinases [6,21,106]. These kinases are generally from three protein kinase families, i.e., AGCIII kinases, CRKs and mitogen-activated protein (MAP) kinases (MPKs) [6]. In the AGCVIII kinases, PID and its paralogs WAVY ROOT GROWTH 1 (WAG1) and WAG2 are distributed on the PM without polarity and directly phosphorylate PIN proteins at three conserved sites, i.e., S1–S3, to facilitate the polar PIN trafficking [64,110]. While FyPP1/3, SAL1 and protein phosphatase 2A (PP2A/RCN1) form a PP6-type phosphatase holoenzyme, which antagonizes the phosphorylation status made by PID [79,111,112]. In cotton, an NF-YB subfamily gene, *GhL1L1*, specifically targeted and activated *GhPP2AA2* to regulate the activity of GhPIN1 during embryonic development [113]. In tomato, PP2C might phosphorylate SlPIN1 to regulate pedicel abscission [114]. Another protein, TOPP4, also acts antagonistically against PID to regulate pavement cell morphogenesis by changing the phosphorylation status of PIN1 [78]. D6PK and its three paralogs D6PK-like (D6PKL) 1–3 from AGCVIII kinases also phosphorylate PIN proteins at S1–S3 together with two additional serine residues, S4 and S5 [115]. PIDs and D6PKs have different phosphosite preferences; PIDs prefer S1–S3; while D6PKs more like the non-conserved phosphosite S4–S5 [115]. D6PK has a polar distribution on the PM in a phospholipid-dependent manner, with the help of phosphatidylinositol 4-phosphate 5-kinases PIP5K1 and PIP5K2 [116]. It localizes on the basal PM, together with the polarly-localized PIN1, PIN2 or PIN4 [76,92]. However, D6PK and PIN2 have different polar localizations in root epidermis cells, suggesting that PIN is not responsible for the polar localization of D6PK [76]. Recently, another AGC-family kinase, PROTEIN KINASE ASSOCIATED WITH BRX (PAX), has been shown to co-localize with PIN proteins and activate PIN-mediated auxin polar transport by timing the development of protophloem sieve element differentiation; meanwhile, BREVIS RADIX (BRX) has strong inhibitory effects on this stimulation [117]. CRK5, which is a PM-localized receptor-like protein kinase, especially phosphorylated the HL of PIN2 to facilitate a gravitropic response in *Arabidopsis* [77]. In *Solanum tuberosum*, calcium-dependent protein kinase 1 (CDPK1), which is regulated by miRNA 390 at the post-transcriptional level, phosphorylates StPIN4 to regulate potato development [118]. The MAP kinase kinase 7 (MKK7)-MPK6 cascade phosphorylates PIN1 at serine 337 (S337) to control shoot branching; this phosphorylation affects the basal localization of PIN1 in xylem parenchyma cells [119]. Besides, phosphorylation of PIN1 at S337/T340 is essential for PIN1 polarity and auxin distribution, and S337/T340 is not directly targeted by PID [120]. Recently, MPK4 and MPK6 have been found to phosphorylate three threonine residues of PIN1, i.e., T227, T248 and T286, these three threonine residues are part of the three TPRXS motifs, which also comprise three highly-conserved serine sites (S1–S3) targeted by PID [66].

For a long time, it was proposed that the phosphorylation of PIN proteins at phosphosites S1–S3 controls the PIN polarity, and the unphosphorylated PIN proteins localize at the basal PM of root cells in the model of PID/WAG-dependent PIN polarity [89,120,121]. However, a recent study showed that both D6PKs and PID/WAGs can activate PIN1 by phosphorylating at the same four serines S1–S4, but PIN1 phosphorylated by D6PKs localizes at the basal PM with Brefeldin A (BFA) sensitive [106]. Thus, the differential effects of D6PKs and PID/WAGs on PIN1 polarity cannot be fully explained by phosphosite preferences [106]. Taking all of this together, three possible mechanisms underlying the effects of PID/WAGs on the PIN polarity have been proposed: Ca^2+^ and the Ca^2+^-dependent proteins TOUCH3 (TCH3), PID-binding protein 1 (PBP1) and the CRK5 pathway; ENP/NPY/MAB4; and peptidyl-prolyl *cis*/*trans* isomerases (Pin1At) together with the MKK/MPK cascade [6].

### 5.3. PIN Protein Subcellular Trafficking and Degradation

The polar distribution of PINs is maintained by continuously, dynamically cycling PIN proteins between the PM and endosomal compartments [21]. ADP-ribosylation factor guanine-nucleotide exchange factors (ARF-GEFs) GNOM, which act at the Golgi apparatus, coordinate the polar distribution of PIN1 [21,122,123]. An ARF family member, ARF1A1C, and a small GTPase RAS GENES FROM RAT BRAINA1b (RabA1b) were shown to recycle PIN proteins to the PM [124,125]. The hydroxylated C24- and C26-acyl-chain sphingolipids are enriched at the *trans*-Golgi network (TGN) subdomains mediating PIN2 polar sorting to the apical membrane of root epithelial cells in *Arabidopsis* [126]. Phospholipase A2 (PLA2), which produces lysophosphatidylethanolamine, has been demonstrated as essential for PIN protein subcellular trafficking to the PM [127]. Further studies have found that choline transporter-like 1 (CTL1) acts at both the secretory and clathrin-coated vesicles of the TGN to regulate trafficking of PIN1 and PIN3 from the TGN to PM through mediating the homeostasis of several membrane lipids, including sphingolipids [128]. In addition to sphingolipid, the balanced sterol composition mediated by sterol methyltransferase 1 (SMT1) also contributes to the polarity of PINs [129]. A recent report pointed out that 14-3-3 epsilon members regulated the polar trafficking of PIN proteins and the recycling processes via endocytosis [130]. More recently, FORKED 1 (FKD1) together with FORKED LIKE 2 (FL2) and FL3 function redundantly to regulate PIN1 asymmetric localization by affecting its secretory pathway in developing veins [131].

The clathrin-mediated endocytosis (CME) plays a crucial role in the internalization of PIN proteins from the PM [132]. Two PI4P 5-kinases, PIP5K1 and PIP5K2, control the minor phospholipid phosphatidylinositol-4,5-bisphosphate (PtdIns(4,5)P2) together to regulate CME for PIN internalization [133]. DRP1A and AP2A1 target PIN1 and PIN2, respectively, to participate in CME for PIN protein recycling, which is essential for the proper distribution of PIN proteins coordinating internal and external developmental signaling [80,81]. The auxin receptor, auxin-binding protein 1 (ABP1), was shown to promote the CME by recruiting clathrin to the PM and mediating the inhibitory effects of auxin on CME [134]. Further study identified Rho-like GTPase 6 (ROP6) and its downstream effector, ROP-interactive CRIB motif-containing protein 1 (RIC1), as the intermediate signaling components between ABP1 and clathrin [135]. However, a recent report confirmed that ABP1 was not a key component in auxin signaling during the developmental processes in *Arabidopsis* [136], suggesting that this model should be carefully re-examined. CATHERIN HEAVY CHAIN 1 (CHC1) and CHC2 are functionally required for PIN proteins’ polarity by internalizing PINs via CME [137], and the CLATHRIN LIGHT CHAIN 2 (CLC2) and CLC3 affect the CHC membrane association, as well as auxin-mediated internalization of PM proteins, including PINs [138]. In addition to clathrin, some other proteins also participate in internalizing PINs through endocytosis, such as ARF-GEF GNOM; ARF-GTPase-activating protein, vascular network defective 3 (VAN3); GNOM-LIKE1 (GNL1); early endosomal components ARF GEF BEN1 (BFA-visualized endocytic trafficking defective1); the Sec1/Munc18 family protein BEN2; and the BIG family ARF GEF BEN3 [139,140,141,142,143,144]. The endocytosis-dependent internalization of PIN2 was reduced in the *S-nitrosoglutathione reductase 1–3* (*gsnor1–3*) mutant, suggesting that NO signaling has negative effects on the internalization of PIN2 [145]. More recently, endogenous NO has been demonstrated to regulate the re-localization of PIN2 in epidermal cells, which is essential for an early gravitropic response in roots [146]. Another negative factor of PIN endocytosis was identified as ALTERED DEVELOPMENT PROGRAM 1 (ADP1); overexpression of *ADP1* inhibited the PIN1 internalization processes [108]. In addition to endocytosis, SEC6/8 and EXO70A1 mediated the exocyst complex involved in auxin polar transport through the recycling of PIN proteins [147,148].

Aside from polar recycling and internalization via CME, the regulation of PIN proteins’ turnover in a ubiquitination-dependent manner in the vacuole also contributes to the PIN polarity at the PM [149]. AUXIN RESISTANT 1 (AXR1), MODULATOR OF PIN 2 (MOP2) and MOP3 were identified as regulators of the stability of PIN proteins [150,151]. Two retromer proteins, sorting nexin 2 (AtSNX2) and vacuolar protein sorting 29 (VPS29), have been shown to regulate the PIN protein turnover by rescuing PIN proteins from degradation and returning them back to their recycling pathways [152,153]. The adaptor protein 3 (AP-3) and the endosomal sorting complex required for transport (ESCRT) participate in the sorting of vacuolar PIN proteins for degradation [154,155,156]. The leaf vein pattern established by PIN1 is regulated by vesicular transport UNH (a VPS51 homolog), which targets PIN1 to a possible destination lytic vacuole for degradation [82]. Interestingly, the application of histone deacetylase inhibitors (HDIs) abolished PIN1 protein without reduced transcripts, suggesting that epigenetic regulation may have a role in controlling the degradation of PIN1 [157]. Additionally, several other regulators have been identified as auxin-regulated genes, participating in auxin-mediated PIN polarity rearrangements in an auxin feedback manner, including a *WRKY* gene member, *WRKY23* and a phosphatidylinositol transfer *PATELLINS* (*PATL*) [158,159]. However, their downstream components in the auxin signaling pathways are yet to be explored.

## 6. Functions of PIN Proteins in Plants

In *Arabidopsis*, the eight *PIN* genes have diverse spatiotemporal dynamic gene expression profiles, differential posttranscriptional regulation manners, as well as invariant protein structures with different subcellular localizations, which result in distinguished functions in development and growth processes mediated by auxin signaling [107,160,161,162,163]. For instance, PIN1 is involved in the auxin basipetal movement, organ initiation, floral bud formation, leaf shape formation, vein patterning, shoot gravitropic responses, as well as shoot vascular development [25,82,160,163,164]. PIN2, which is mainly expressed in cortical and epidermal cells of root-tip elongation zones and embryogenesis, has been demonstrated to be involved in basipetal transport and gravitropism [77,162,164,165,166,167]. PIN3 participates in the formation of the lateral root at the early steps, apical hook formation and maintenance, as well as gravitropic and phototropic responses [99,108,168,169]. Furthermore, PIN3 has been demonstrated to play a role in the ratio of red light- and far red light-induced shade avoidance and the reduction of lateral root density, which closely links to fitness under the competition for light [170,171]. Similarly to PIN3, PIN4 also has a role in phototropic response and apical hook development [108,172]. PIN4, which expresses in the meristems of developing and mature roots, facilitates the sink-driven auxin gradients below the quiescent center to regulate root patterning [161]. PIN5, which localizes at the ER, participates in a series of auxin-related developmental processes, including lateral root initiation, cotyledon expansion, early embryogenesis, root and hypocotyl growth, by mediating the subcellular compartmentalization of auxin [60]. PIN6 has a dual localization at the PM and ER to regulate both auxin transport across the PM and intracellular auxin homeostasis [62]. Further study has shown that the dual localization of PIN6 is determined by phosphorylation, and the subcellular location of PIN6 fine tunes the bolting [61]. PIN6 also has a broad role in auxin-signaling-mediated developmental processes, such as the lateral/adventitious root organogenesis, root waving, primary/lateral root development and growth, root hair outgrowth and shoot apical dominance [62,173,174]. PIN7 is also a mediator of the gravitropic response, which negatively controls the radial growth of root systems [175]. PIN8 is an ER-localized protein that regulates subcellular auxin homoeostasis, which controls the development processes of pollen, male gametophyte and the sporophyte [176,177].

In addition to *Arabidopsis*, some progress addressing the important roles of these auxin efflux carriers in auxin-mediated processes of development and growth has been made in some other plant species. In a plant close to *Arabidopsis*, *Cardamine hirsuta*, PIN1 was involved in promoting leaflet initiation [32]. In maize, ZmPIN1 proteins were required for cell and tissue differentiation during maize embryogenesis and endosperm development, as well as post-embryonic vegetative and reproductive development [178]. In *Solanum lycopersicum*, SlPIN1 negatively controlled follower abscission by regulating auxin accumulation at the ovary and abscission zone [114]. The co-silencing of *SlPIN 4* and *5* changes the shoot architecture to have a large angle between the base and the shoot apical meristem, but did not affect fruit development [52]. In *Nicotiana tabacum*, *NtPIN4* was induced by auxin and involved in auxin-dependent branching by negatively regulating the growth of axillary bud [46]. In *Medicago truncatula*, PINs have a role in forming the root nodules in an auxin-dependent manner by silencing *MtPIN2*/*3*/*4*, which reduced the number of nodules [44,45,179]. In cotton, PIN proteins have a role in regulating fiber growth, and the heterologous overexpression of *GhPIN1a_Dt*, *GhPIN6_At* and *GhPIN8_At* in *Arabidopsis* changed the number and size of leaf trichomes [41]. In rice, *OsPIN2* was expressed in epidermal and cortex cells of roots, and it regulated the gravitropic responses and root system architecture [180]. Overexpression of *OsPIN2* led to increased aluminum internalization by enhancing the endocytic vesicular trafficking capacity in root apex [181].

In woody plants, overexpression of *PtPIN9* from *Populus tremula* × *Populus alba* promoted lateral root formation [182]. Meanwhile, overexpression of *PtoPIN3a* led to the observation of a shrunken leaf phenotype, suggesting that this gene is involved in *Populus* leaf morphogenesis [28]. In apple, the reduced activities of natural allelic *MdPIN1b* were proposed to be responsible for the dwarfing tree architecture phenotype in Malling 9 rootstock [35]. In *Malus* × *domestic* Royal Gala, heterologous overexpression of *MdPIN1* in *Arabidopsis* led to changes in root architecture with enhanced phototropic and geotropic responses [34].

## 7. Conclusions and Perspectives

To date, much progress has been made in understanding the roles of PIN proteins on developmental and growth processes. The activities of PIN proteins are fine-tuned at multiple layers, including transcriptional regulations, post-transcriptional modifications for subcellular trafficking, recycling, endocytosis and vacuolar trafficking for degradation. The identification of PIN proteins in diverse plant species led us to update our understanding of the evolutionary history of PIN proteins in the plant kingdom. However, the evolutionary history of PIN proteins is still in debate. Extensive work has been done to draw a comprehensive regulatory network of PIN proteins, and posttranslational modifications beyond phosphorylation that regulate PIN proteins and their distribution might be brought under the spotlight soon, since information regarding these aspects has barely been reported so far. Although three possible mechanisms interpreting the different effects of PID/WAGs on the PIN polarity have been proposed, the exact mechanisms still remain unclear. To achieve this goal, genetic methods combined with the computational analysis of large-scale datasets, such as transcriptome, proteomics and metabolome, may contribute to identifying new regulatory layers and the associated candidates. The successful identification of *WRKY23* and *PATL* as the regulators of PIN polarity rearrangements from transcriptome datasets has shown the power of this approach [158,159]. Thus, the co-expression network and the protein interacting network of PIN proteins presented here might provide a range of potential candidates regulating PIN actions, which await discovery. These advanced techniques have allowed for the more precise dissection of the dynamic localization of PIN proteins in the PM. This leads to the renewal of our understanding of the mechanisms of phosphorylation underlying PIN protein polar localizations. Moreover, the methods established regarding the PIN proteins could be valuable toolkits to dissect the regulatory machinery that controls the polarity of other PM proteins and find the potential key regulators involved in these processes. Although our understanding of PIN proteins has been based on the model plant *Arabidopsis*, the knowledge gained from this could facilitate the discovery of these processes in other plants with new features.

## Figures and Tables

**Table 1:** Summary of *PIN* genes in 31 plants found by genome-wide analysis.

Species	No. of Predicted Loci ^a^	No. of *PIN* Genes	References
*Arabidopsis thaliana*	27,416	8	[17]
*Arabidopsis lyrata*	32,670	8	[17,36]
*Aquilegia caerulea*	-	6	[36]
*Brachypodium distachyon*	31,694	11	[17,36]
*Brassica rapa*	40,492	15	[37]
*Carica papaya*	24,782	6	[38]
*Capsicum annuum*	34,899	10	[39]
*Citrullus lanatus*	23,440	11	[40]
*Glycine max*	56,044	23	[26,27]
*Gossypium arboreum*	41,330	12	[41]
*Gossypium hirsutum*	66,577	17	[41,42]
*Gossypium raimondii*	37,505	10	[41]
*Lotus japonicus*	42,399	11	[27,43]
*Marchantia polymorpha*	19,287	4	[36]
*Medicago truncatula*	50,894	11	[44,45]
*Mimulus guttatus*	28,140	10	[36]
*Nicotiana tabacum*	-	20	[46]
*Nicotiana sylvestris*	-	11	[46]
*Nicotiana tomentosiformis*	-	12	[46]
*Oryza sativa*	39,049	12	[36,47,48]
*Phaseolus vulgaris*	27,082	16	[27]
*Phyllostachys heterocycla*	31,987	14	[49]
*Physcomitrella patens*	26,610	5	[36]
*Populus trichocarpa*	41,335	15	[28,50]
*Selaginella moellendorffii*	22,273	9	[29]
*Setaria italica*	34,584	12	[51]
*Solanum lycopersicum*	34,727	10	[52]
*Sorghum bicolor*	34,129	11	[53]
*Solanum tuberosum*	39,031	10	[54]
*Vitis vinifera*	26,346	8	[17,29]
*Zea mays*	63,480	15	[55,56]

^a^ The data were from Phytozome (available online: https://phytozome.jgi.doe.gov/pz/portal.html/) and the Plant Genome Duplication Database (PGDD, available online: http://chibba.agtec.uga.edu/duplication/) [57].

## Supplementary Materials

Supplementary materials can be found at http://www.mdpi.com/1422-0067/19/9/2759/s1.
